# Natural Clinoptilolite as a Functional Mineral Component in Alginate Hybrid Microcapsules for Controlled Amoxicillin Release

**DOI:** 10.3390/pharmaceutics18070878

**Published:** 2026-07-17

**Authors:** İrem Toprakçı, Ebru Kurtulbaş, Dorina Simedru, Anca Becze, Oana Cadar, Selin Şahin

**Affiliations:** 1 Department of Chemical Engineering, Faculty of Engineering, Istanbul University-Cerrahpaşa, Avcılar, 34320 Istanbul, Türkiye; irem.toprakciyuksel@iuc.edu.tr (İ.T.); ebru.kurtulbas@iuc.edu.tr (E.K.); 2 INCDO-INOE 2000, Research Institute for Analytical Instrumentation, 67 Donath Street, 400293 Cluj-Napoca, Romania; dorina.simedru@icia.ro (D.S.); anca.becze@icia.ro (A.B.)

**Keywords:** clinoptilolite, hybrid microcapsules, inorganic–organic composites, amoxicillin, release kinetics

## Abstract

**Background/Objectives:** Natural clinoptilolite–amoxicillin hybrids (CNZ@AMOX) were incorporated into alginate microcapsules via ionic gelation to develop a hybrid mineral–polymer delivery system for the controlled release of amoxicillin. **Methods:** A face-centered central composite design combined with response surface methodology (FCCD-RSM) was utilized to assess the effects of the zeolite/sodium alginate ratio, alginate concentration, calcium chloride concentration and curing time on the encapsulation efficiency (EE), sphericity factor (SF), and roundness (Rn). **Results:** The EE ranged from 5.9% to 91.3%, depending on the formulation composition. Numerical optimization identified the optimal conditions as 70.962% EE, 0.05 SF and 1.00 Rn, with a desirability score of 0.873. The incorporation of natural clinoptilolite improved microcapsule structural integrity and reduced the initial burst release by modulating diffusion pathways within the hybrid matrix. The optimized CNZ@AMOX exhibited pH-dependent release behavior, with minimal drug release in simulated gastric fluid (SGF) and diffusion-controlled release in simulated intestinal fluid (SIF), which was best described by the Korsmeyer–Peppas model. **Conclusions:** These findings demonstrate that zeolite–alginate hybrid microcapsules represent promising inorganic–organic composite carriers for the pH-responsive and controlled delivery of AMOX.

## 1. Introduction

Oral delivery of antibiotics such as amoxicillin (AMOX) remains challenging because of premature drug release under gastric conditions [1]. Moreover, the delivery efficiency may be limited by many factors such as low drug solubility and/or permeability, gastrointestinal instability, and reduced antibacterial activity [2]. The potential adverse effects of these drugs on the gastric mucosa should also be considered, as they may lead to gastric ulcers [3]. The targeted delivery of pharmacologically active compounds to specific sites in the human body remains a significant challenge [4]. Therefore, there is a growing demand for effective therapies with fewer adverse effects that limit their application. Microencapsulation can protect these substances from physicochemical, chemical, and mechanical environmental effects [5].

Clinoptilolite, as one of the most abundant natural zeolites, has attracted considerable interest in biomedical research because of its unique physicochemical characteristics [6]. The naturally occurring zeolite clinoptilolite has been widely investigated, owing to its structural stability, ion-exchange capacity, and adsorption efficiency [7]. Its porous aluminosilicate framework, high specific surface area, and adsorption/ion-exchange capabilities, make clinoptilolite a promising inorganic carrier for drug delivery. These characteristics enable efficient drug binding and may modulate release profiles [8]. However, the direct use of drug-loaded zeolites may be hindered by handling difficulties, dispersion instability, and an initial burst release in aqueous media [9]. Therefore, incorporating clinoptilolite–drug hybrids into polymeric matrices may overcome these limitations by providing an additional diffusion barrier and improving processability. Clinoptilolite-based molecular sieves have been widely used as drug-binding agents in alginate composite hydrogels to enhance mechanical integrity and modulate pH-responsive release behavior [10].

Alginate-based composite microcapsules have received attention from the pharmaceutical, food science, and biomedical engineering communities due to their biocompatibility, biodegradability and capacity for ionic gelation under mild conditions [11,12]. Therefore, alginate gel beads incorporating porous zeolite fillers have been proposed as effective carriers to improve carrier performance [13].

On the other hand, the performance of alginate-based composite microcapsules is highly sensitive to gelation conditions. Parameters such as polymer and crosslinker concentrations, inorganic/polymer ratio and curing time affect crosslinking density, droplet formation behavior and particle geometry [14]. Therefore, controlling both encapsulation efficiency and particle geometry is essential for designing robust oral delivery systems. Response surface methodology provides a systematic framework to identify optimal conditions with reduced experimental burden compared to one-factor-at-a-time approaches [15,16,17].

Natural clinoptilolite was selected for this study owing to its intrinsic non-toxicity, excellent thermal stability, chemical tunability, biocompatibility, as well as ability to adsorb diverse molecules within its micro- and mesoporous structure [18]. In this study, calcium–alginate microcapsules incorporating clinoptilolite–amoxicillin hybrids (CNZ@AMOX) were developed by optimizing the ionic gelation process using a face-centered central composite design (FCCD-RSM). The effects of zeolite/sodium alginate ratio, sodium alginate concentration, calcium chloride concentration and curing time were evaluated in relation to encapsulation efficiency (EE), sphericity factor (SF) and roundness (Rn). In parallel with process optimization, the CNZ@AMOX hybrid was characterized using X-ray diffraction (XRD) and scanning electron microscopy (SEM). Finally, the contribution of clinoptilolite to the pH-dependent release behavior of AMOX was assessed under simulated gastrointestinal conditions, while the release kinetics were analyzed using established mathematical models.

## 2. Materials and Methods

### 2.1. Materials

Amoxicillin trihydrate (AMOX, ≥98% purity) was provided by Santa Cruz Biotechnology (Santa Cruz, CA, USA). Phosphate-buffered saline, sodium alginate as biopolymeric matrix and calcium chloride (CaCl_2_) as crosslinker were purchased from Sigma-Aldrich (St. Louis, MO, USA). According to the manufacturer’s specifications, the sodium alginate exhibited a viscosity of 20–400 cP for a 1% aqueous solution. Since sodium alginate is a naturally derived polydisperse polymer, an exact molecular weight is not specified by the manufacturer.

Natural clinoptilolite-rich zeolite, CNZ, was obtained and characterized as previously reported [19]. The natural zeolite was collected from the Chilioara open-pit mine in Salaj County, Romania. The raw mineral was first ground and sieved using a vibratory disk mill RS 200 (Retsch, Haan, Germany) to obtain particles smaller than 1 mm, and then dried at 105 °C. The powder was subsequently micronized to below 40 µm using a PilotMill-2 system (PilotMill-2 FPS1015, Como, Italy) and thermally activated at 400 °C for 4 h in air to improve adsorption efficiency toward organic species, particularly pharmaceutical compounds, while ensuring sterilization.

### 2.2. Preparation of CNZ@AMOX

2.5 g of CNZ was dispersed in 500 mL of an aqueous AMOX solution (250 mg); the pH of the suspension was adjusted to 7.4 and the mixture was magnetically stirred at room temperature. At selected time intervals (10, 20, 30, 45, 60, 90, 120, 180, 240, 300, 360, 420 and 480 min), 1 mL aliquots of the supernatant were withdrawn, filtered and analyzed. The concentration of AMOX was determined using a Vanquish ultra-high-pressure liquid chromatography (HPLC) system (Thermo Scientific, Germering, Germany) equipped with a diode array detector (DAD HL, Dionex, Germering, Germany) [20]. After the completion of the adsorption process, the solid phase was recovered by vacuum filtration through a 0.45 μm cellulose membrane, thoroughly washed with ultrapure water (Purelab flex 3 system, Elga Labwater, Buckinghamshire, UK) to remove unbound drug, and dried at 40 °C to constant weight.

### 2.3. Characterization of CNZ@AMOX

The X-ray diffraction (XRD) patterns were recorded using a D8 Advance (Bruker, Karlsruhe, Germany) diffractometer with CuKα radiation (λ = 1.54060 Å), operating at 40 kV and 40 mA. The morphology was examined using a scanning electron microscope (VEGAS 3 SBU, Tescan, Brno-Kohoutovice, Czech Republic) with an EDX detector (Quantax EDS, Bruker, Karlsruhe, Germany).

### 2.4. Preparation of CNZ@AMOX–Alginate Microcapsules by Ionic Gelation

The external ionic gelation method was used to prepare CNZ@AMOX–alginate microcapsules. Alginate solutions at concentrations of 1%, 1.5% and 2% (*w*/*v*) were prepared using a magnetic stirrer (Model MSH-20D, DAIHAN Scientific Co. Ltd., Wonju-si, Gangwon-do, Republic of Korea). Subsequently, CNZ@AMOX were added to these solutions in varying proportions (1/20, 1/10 and 2/10, *w*/*v*) as determined by the experimental design methodology. The mixtures were stirred for 30 min to ensure homogeneity. Then, the resulting drug–zeolite–alginate dispersion was dropped into calcium chloride solutions prepared at concentrations of 2%, 8.5% and 15% (*w*/*v*) (Table S1) using a syringe pump (New Era Pump Systems Inc., Farmingdale, NY, USA) in order to maintain a constant flow rate (3.5 mL/min). Figure S1 illustrates the general workflow of this encapsulation step. The resulting microcapsules were stirred on a magnetic stirrer for 10, 20 or 30 min, according to the predetermined experimental design. Following crosslinking, the microcapsules were washed 3 times with ultrapure water, filtered and dried under a fume hood for 3 days.

### 2.5. Determination of Encapsulation Efficiency

The AMOX encapsulated in the CNZ@AMOX–alginate microcapsules was extracted by dispersing the beads in phosphate-buffered saline using a homogenizer (IKA T25, ULTRA-TURRAX, Staufen, Germany). The encapsulation efficiency (EE) was determined by quantifying the concentration of AMOX in the dispersion medium and calculated according to the method described by Kalam et al. [21]:

This is example 1 of an equation:

(1) $$EE\;(\%)=\frac{W_{1}-W_{2}}{W_{1}}\times 100$$

where W_1_ = Total actual amount of AMOX used in the formulation, and W_2_ = Amount of free AMOX analyzed in the supernatant.

The concentration of the AMOX was determined chromatographically by means of HPLC (Table S2).

### 2.6. Physical Characterization of CNZ@AMOX–Alginate Microcapsules

The physical characterization of the CNZ@AMOX–alginate microcapsules was performed using an optical microscope equipped with a CMOS-based digital imaging system (Cameram 5, SOIF Optical Instruments Co., Ltd., Shanghai, China) attached to an optical microscope. The particle dimensions (d_min_ and d_max_), projected area, perimeter, sphericity factor (SF), and roundness (Rn) were determined from the microscopic images to evaluate the size and morphology of the microcapsules. The camera has a 5-megapixel sensor (2592 × 1944 px) and is equipped with built-in analysis software. It enables dimensional measurements (d_min_, d_max_, area and perimeter) and morphological evaluation (roundness and sphericity factor) [22]:

(2) $$Sphericty\;factor\;\left(SF\right)=\;\frac{d_{max}-d_{min}}{d_{max}+d_{min}}$$

(3) $$Roundness\;(Rn)=\frac{P^{2}}{4\pi A}$$

### 2.7. Statistical Analysis

A face-centered central composite design combined with response surface methodology was applied to the ionic gelation process as a three-level factorial design to optimize four parameters. Furthermore, this approach provides an evaluation of the effects of the process parameters and their interactions on the relevant system. In this study, Design-Expert software (Version 12.0.1.0, Stat-Ease Inc., Minneapolis, MN, USA) was used. The analysis of variance (ANOVA) test was performed to assess the model fit and determine interactions between the variables using the same software. Additionally, Pareto charts showing the standardized effects of the independent variables on the response parameters were generated using Minitab statistical software (Minitab^®^, Version 22, Minitab LLC, State College, PA, USA).

The variables and their levels (Table S1) used in the experimental design were determined based on literature [23,24] and the results obtained from our earlier experiments [25,26,27,28].

### 2.8. In Vitro Drug Release Under Simulated Gastrointestinal Conditions

The in vitro release behavior of AMOX from CNZ@AMOX–alginate microcapsules was evaluated under simulated gastrointestinal conditions using simulated gastric fluid (SGF, pH 1.2) and simulated intestinal fluid (SIF, pH 6.8). The microcapsules (0.2 g) were placed in 50 mL of SGF. The mixture was then incubated at 37 ± 0.5 °C under gentle agitation (100 rpm) using a shaking incubator. After 2 h, the microcapsules were separated and transferred into fresh SIF (50 mL) to simulate intestinal conditions. At predetermined time intervals, aliquots (1 mL) were withdrawn and filtered using a 0.45 µm membrane filter. The withdrawn volume was immediately replaced with an equal volume of fresh release medium to maintain sink conditions. The concentration of released AMOX was determined using HPLC under the chromatographic conditions described in Table S2.

#### Mathematical Modeling of Drug Release Kinetics

Various kinetic models, including the zero-order (Equation (4)), first-order (Equation (5)), Higuchi (Equation (6)) and Korsmeyer–Peppas (Equation (7)) models, were applied to investigate the drug release mechanism from CNZ@AMOX–alginate microcapsules [29,30].

(4) $$Q_{t}=Q_{0}+k_{0}t$$

where Q_0_ is the initial concentration of the drug in the solution (mg g^−1^), Q_t_ is the concentration of the drug released at time t (mg g^−1^), k_0_ is the zero order release constant (mg min^−1^), and t is time (min).

(5) $$\log C_{t}=\log C_{0}-k_{1}t$$

where C_0_ is the initial concentration of the drug in the microcapsules (mg g^−1^), C_t_ is the concentration of the drug remaining in the microcapsules at time t (mg g^−1^), and k_1_ is the first order release constant (min^−1^).

(6) Qt=kHt12

where k_H_ is the Higuchi release constant (mg min^−1/2^).

(7) $$\frac{M_{t}}{M_{\infty }}=K\;t^{n}$$

where M_t_ is the amount of drug released at time t (mg g^−1^), M_∞_ is the total amount of drug released at infinite time (maximum release) (mg g^−1^), K is the release rate constant (min^−n^), and n is the release exponent indicating the release mechanism.

## 3. Results and Discussion

### 3.1. Preparation and Characterization of Clinoptilolite–Amoxicillin Hybrid (CNZ@AMOX)

**Figure 1:**
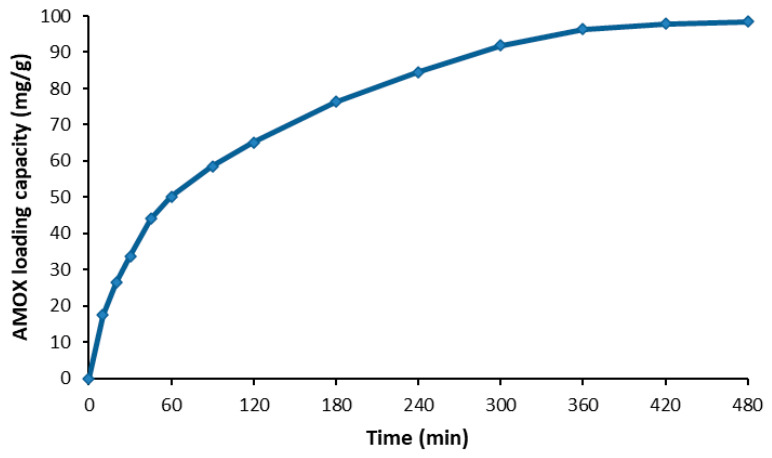
Adsorption kinetics of amoxicillin onto the CNZ as a function of contact time.

The amount of AMOX adsorbed onto the CNZ sample at different contact times was determined by HPLC (Figure 1). The equilibrium was attained after 480 min, with an adsorption capacity of approximately 50%. This moderate drug uptake is consistent with the known limitations of unmodified natural zeolites, whose negatively charged framework is balanced by hydrated inorganic cations (Ca^2+^, Mg^2+^, Na^+^, and K^+^), resulting in a predominantly hydrophilic character and poor affinity for hydrophobic or anionic species [18]. Under near-neutral conditions, AMOX exists mainly in its anionic form at pH > 7.4, which may induce electrostatic repulsion from the clinoptilolite surface. Thus, the observed adsorption behavior can be attributed to the interplay between surface hydrophilicity, competition at exchangeable sites, and AMOX speciation in the solution [31]. The adsorption of AMOX onto heat-treated natural zeolite followed pseudo-second-order kinetics (PSO, R^2^ = 0.996), with a calculated equilibrium capacity of 113.6 mg/g, indicating chemisorption as the rate-controlling mechanism [32].

**Figure 2:**
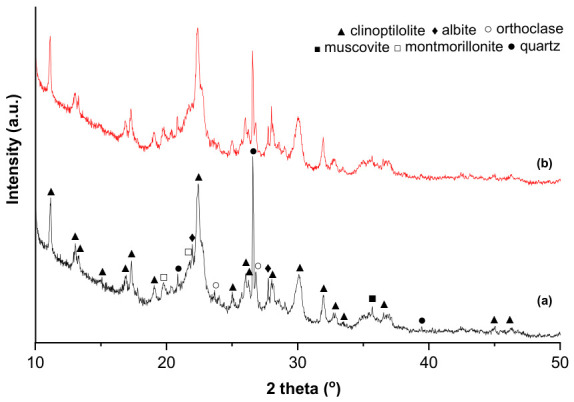
XRD patterns of (**a**) CNZ and (**b**) CNZ@AMOX samples.

The XRD patterns of CNZ and CNZ@AMOX samples (Figure 2) confirm clinoptilolite (PDF card No. 01-080-1557) as the predominant crystalline phase, as evidenced by the characteristic diffraction peaks of the clinoptilolite zeolite framework [19,33]. Secondary mineral phases were also identified, including albite (PDF card No. 00-020-0548), orthoclase (PDF card No. 00-031-0966), montmorillonite (PDF card No. 00-058-2038), muscovite (PDF card No. 00-060-1516), and quartz (PDF card No. 01-070-7344). The semi-quantitative phase analysis using the reference intensity ratio (RIR) method suggested a clinoptilolite content of ~65%. The overall crystallinity was approximately 55% for both samples; an amorphous fraction attributed to volcanic glass was evidenced by a broad diffraction halo centered at approximately 2θ = 25° [34]. XRD analysis revealed that antibiotic loading did not induce observable changes in the diffraction pattern of clinoptilolite, since both peak positions and relative intensities remained essentially unchanged. No crystalline transformation or additional phase formation occurred, indicating the structural stability of the zeolitic framework after functionalization.

**Figure 3:**
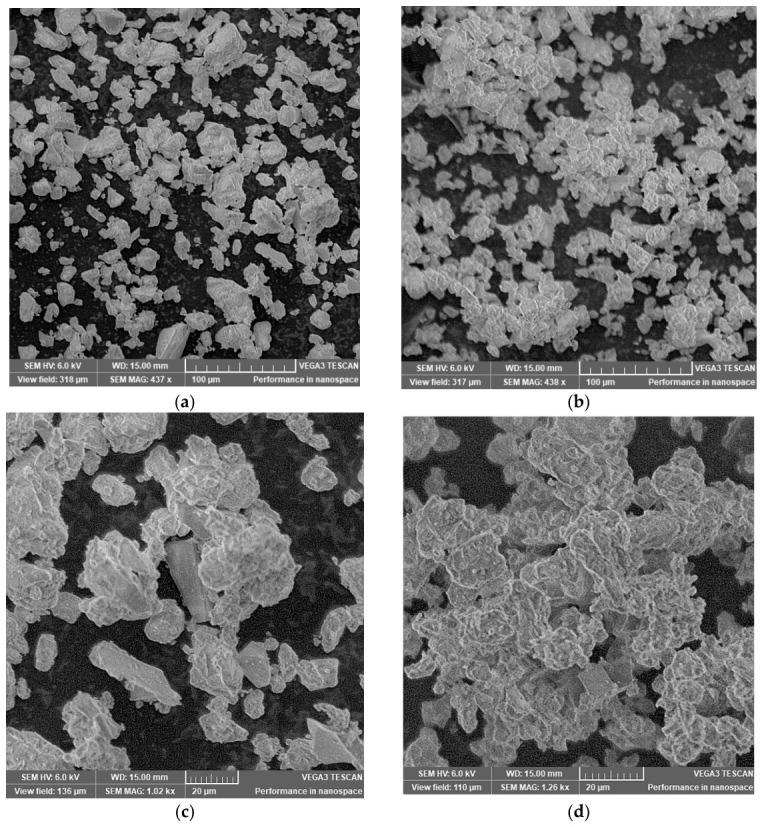
SEM images of (**a**) CNZ and (**b**) CNZ@AMOX samples at low magnification; (**c**) CNZ and (**d**) CNZ@AMOX samples at high magnification.

The CNZ sample (Figure 3a) displays an irregular and highly porous structure consisting of weakly aggregated particles with rough surfaces. These particles vary in size and shape, resulting in a heterogeneous structure. AMOX loading promotes particle agglomeration without causing significant changes in particle appearance, even at high magnifications (~1.20 kX). EDX analysis reveals similar elemental compositions (Si, Al, Ca, K based) for both samples, indicating that the addition of AMOX did not change the elemental composition of the particles and suggesting effective incorporation of AMOX. These observations are in good agreement with the XRD results.

### 3.2. Face-Centered Central Composite Design-Based Microcapsules Prepared by Ionic Gelation

Microencapsulation was performed by ionic gelation using sodium alginate following the preparation of CNZ@AMOX hybrids. The FCCD experimental design matrix and corresponding responses for EE, SF and Rn are presented in Table 1.

Thirty experimental runs, including seven center-point replicates, were performed. The design variables selected in this study (polymer concentration, Ca^2+^ crosslinker concentration, and processing time) are consistent with previous ionic gelation studies, which identify alginate and CaCl_2_ concentrations as primary determinants of bead characteristics, including encapsulation and morphology [10,35,36]. EE ranged from 5.9% to 91.3%, indicating a strong dependence on formulation composition and ionic gelation conditions. This broad response-range supports the suitability of FCCD for modeling and optimizing microcapsules. The highest EE values (>90%) were achieved at low zeolite/sodium alginate ratios combined with high CaCl_2_ concentrations, highlighting the role of Ca^2+^-mediated crosslinking density in enhancing encapsulation performance. Higher CaCl_2_ concentrations promote the formation of a denser “egg-box” network that can reduce drug diffusion into the gelling bath [37]. This trend is consistent with the factorial design findings of Sarangi et al. [35], who reported improved entrapment with increasing CaCl_2_ concentration due to the formation of a denser ionic network.

The obtained EE values are comparable to those reported in previous studies of AMOX encapsulation using biopolymeric carriers. Angadi et al. reported EE values of 52–92% for AMOX-loaded sodium alginate microbeads coated with chitosan [38,39]. Girigoswami reported an encapsulation efficiency of 64% for AMOX in chitosan–alginate nanohydrogels [39]. EE values of 78.4% and 85.2% were reported for pectin–alginate–hydroxypropyl methylcellulose beads prepared using CaCl_2_ and FeCl_3_, respectively [40]. AMOX-loaded alginate beads containing poloxamer 407 achieved an EE of 85.74% [41]. Similarly, the highest EE values for prednisolone-loaded alginate beads prepared by ionic gelation were obtained at higher alginate and CaCl_2_ concentrations, supporting the central role of these variables in encapsulation performance [36].

SF values ranged from 0.01 to 0.54, as shown in Table 1. Lower SF values, corresponding to more spherical microcapsules, were generally obtained at moderate alginate concentrations and high CaCl_2_ levels. Morphology is influenced by several interacting variables, including polymer viscosity, curing conditions, and the crosslinking environment [36]. Increased polymer viscosity combined with stronger ionic crosslinking may promote the formation of mechanically stable, uniformly shaped beads [42]. A similar trend has been reported for alginate gel beads, in which increasing CaCl_2_ concentration caused shrinkage of the gel network and reduced bead size because of the higher crosslinking density [13].

Table 1 also indicates that the roundness values ranged from 0.48 to 3.46. In alginate bead studies, roundness values generally range from 0 to 1, with 1 representing a perfect circle [43]. Roundness was calculated using the perimeter-area relationship (Equation (3)), whereas increasing Rn values indicated progressively greater shape irregularity. Therefore, Rn was set to a target value of 1 during numerical optimization to promote the formation of near-spherical microcapsules. This approach is consistent with previous ionic gelation studies reporting high circularity for alginate beads, reflecting their tendency to form near-spherical particles under appropriate formulation and processing conditions [44]. Moreover, composite alginate systems incorporating whey protein aggregates have been reported to further improve bead morphology and structural integrity, highlighting the importance of matrix organization in producing stable and well-defined spherical particles [45].

### 3.3. Effect of Process Variables on Encapsulation Efficiency

Table 2 shows that formulation and processing variables have a considerable influence on encapsulation efficiency. The model’s significance (*p* < 0.0001), high coefficient of determination (R^2^ = 0.9917) and non-significant lack of fit (*p* > 0.05) show a good agreement between experimental and predicted values. Model adequacy was evaluated using the adjusted R^2^ and predicted R^2^ values as well [46]. The close agreement between these values (difference < 0.2) for all responses confirms the reliability and predictive capability of the models [47].

Among the main effects, the zeolite/sodium alginate ratio (A) and calcium chloride concentration (C) were the most influential parameters affecting EE (*p* < 0.0001), whereas sodium alginate concentration alone (B) showed no statistically significant linear effect (*p* = 0.8208), as shown in Table 2. Processing time (D) had no significant influence (*p* = 0.0605). Significant interaction terms (AC and BD) and quadratic effects (A^2^, B^2^, C^2^ and D^2^) revealed a pronounced nonlinear response behavior. The polynomial equation (Equation (8)) further supports the nonlinear effects of the studied variables on EE.

EE (%) = +29.92 − 20.17 A − 0.1606 B + 15.18 C − 1.41 D + 1.41 AB − 9.00 AC + 3.07 AD  + 0.5994 BC − 6.25 BD + 3.47 CD + 25.43 A^2^ − 10.21 B^2^ − 4.10 C^2^ − 3.04 D^2^ (8)

**Figure 4:**
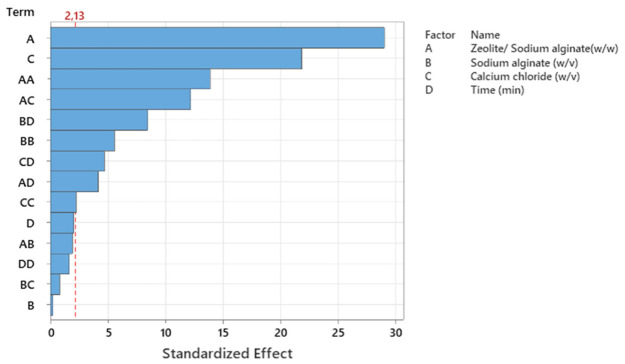
Pareto chart showing the standardized effects of process variables on encapsulation efficiency (α = 0.05).

Pareto analysis (Figure 4) confirmed that CaCl_2_ concentration (C) and the zeolite/alginate ratio (A) have the strongest effects on encapsulation efficiency, as both effects clearly exceeded the significance threshold (α = 0.05). These effects were followed by their quadratic terms (A^2^) and their interaction of them (AC). This tendency indicates a pronounced nonlinear dependence of EE on the formulation variables, whereas sodium alginate concentration (B) and processing time (D) exhibited comparatively minor effects.

**Figure 5:**
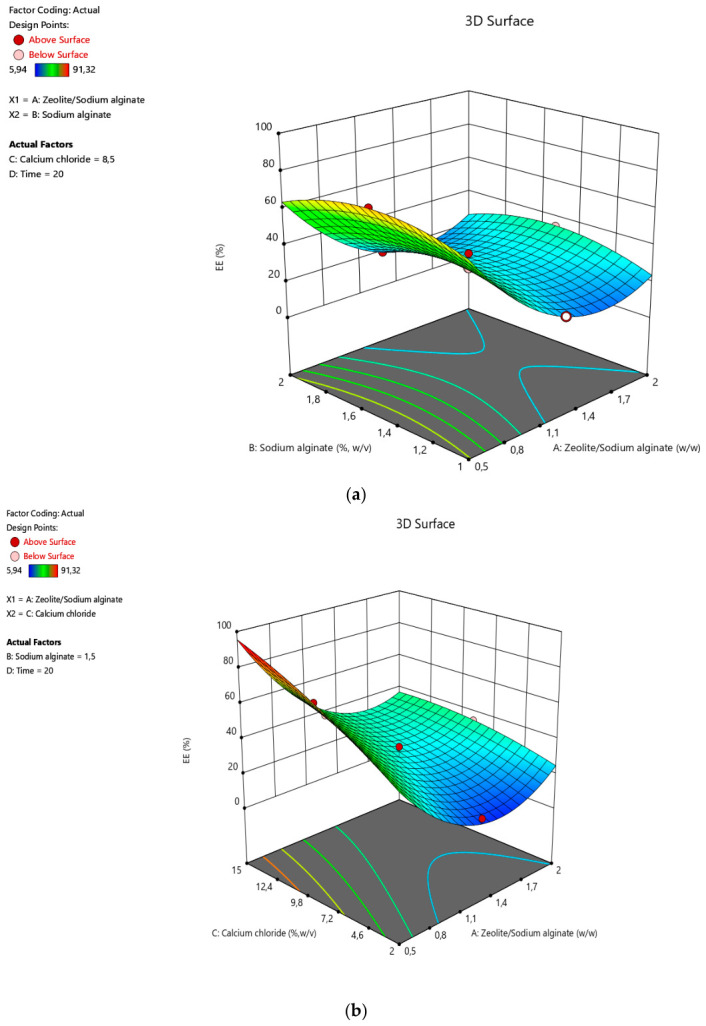

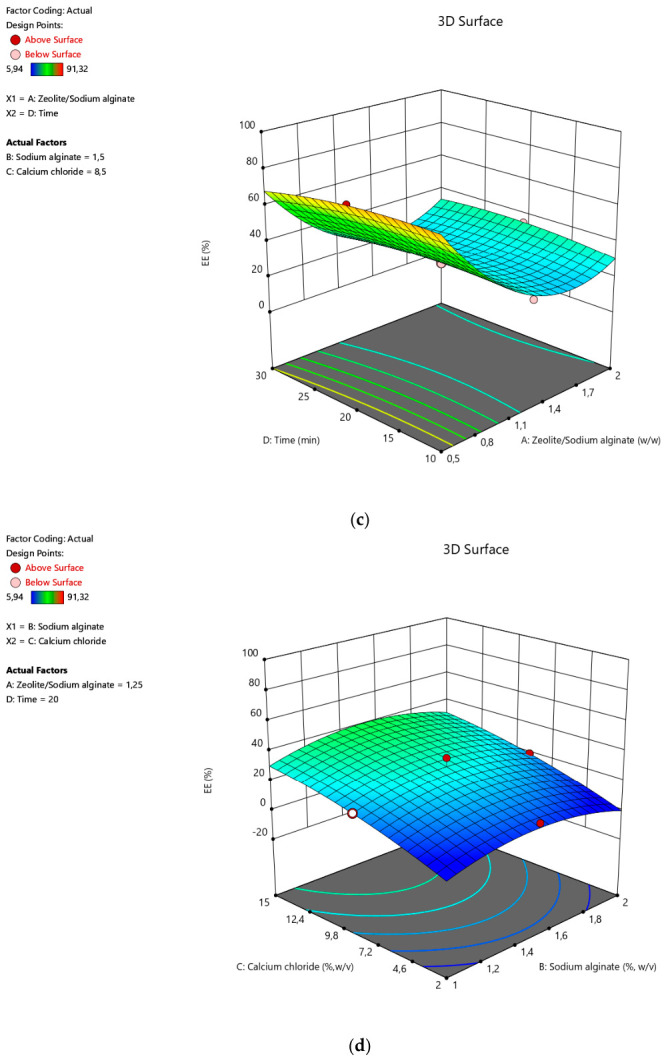
Three-dimensional response surface plots showing the interactive effects of zeolite/sodium alginate and sodium alginate (**a**), zeolite/sodium alginate and calcium chloride (**b**), zeolite/sodium alginate and time (**c**), and sodium alginate and calcium chloride (**d**) on encapsulation efficiency of CNZ@AMOX–alginate microcapsules.

The three-dimensional response surface plots (Figure 5) show the interactive effects of the variables on encapsulation efficiency. As shown in Figure 5a, the zeolite/sodium alginate ratio exhibited a strong influence on EE. The curvature of the surface supports the significant quadratic effect of the zeolite/sodium alginate ratio (A^2^). On the other hand, sodium alginate concentration had no significant linear contribution, consistent with the ANOVA (Table 2) and Pareto (Figure 4) analyses. The strong effect of the zeolite/sodium alginate ratio demonstrates the importance of balancing the inorganic component and polymer matrix in the microcapsule systems. At lower zeolite/alginate ratios, sufficient polymer may provide effective coating of the CNZ@AMOX hybrids, leading to higher encapsulation efficiency. However, excessive zeolite loading in the composite may disrupt polymer continuity and create diffusion pathways through which the active material can escape [48]. Although this principle has mainly been discussed for miniemulsion-based systems, the general dependence of hybrid morphology on component compatibility may also explain the coupled effects observed in polymer–inorganic composites [49]. In such systems, formulation variables influence performance by governing the structural organization and compatibility between the organic matrix and the inorganic phase [50].

Figure 5b demonstrates a strong synergistic interaction between zeolite/sodium alginate ratio and CaCl_2_ concentration, where increasing CaCl_2_ concentration at lower zeolite/sodium alginate ratio levels enhanced EE markedly. This interaction is consistent with the significant AC term identified in the regression model (Equation (8)) and Pareto chart (Figure 4), showing the critical role of crosslinker (CaCl_2_) concentration in alginate network formation. Since alginate network contraction depends on both polymer availability and calcium ion penetration, crosslinker concentration naturally interacts with formulation composition [16].

Figure 5c also confirms the non-significant main effect of time as observed in the statistical analysis earlier (Table 2 and Figure 4). Figure 5d shows a smooth response surface with limited curvature, consistent with the relatively small standardized effects of the BC and B^2^ terms observed in the Pareto analysis. In external gelation, alginate gelation is rapid. Therefore, extending gelation time beyond the initial stage often has a limited effect on the responses and mainly reflects diffusion-controlled curing [51]. A previous study of Ca-alginate beads reported that an optimal hardening time of approximately 30 min was sufficient to establish a stable crosslinked network, while longer curing times mainly promote further densification without enhancing encapsulation efficiency [52]. Kim et al. similarly reported that heating temperature significantly influenced rupture strength and size in thermally treated calcium-alginate beads, whereas heating time had no significant linear effect on the selected responses [53].

### 3.4. Effect of Process Variables on Sphericity Factor and Roundness

Table 2 shows that both the sphericity factor and roundness were significantly influenced by the independent variables with highly significant regression models (*p* < 0.0001), high coefficients of determination (R^2^ > 0.98) and non-significant lack of fit (*p* > 0.05). In addition, the close agreement between adjusted and predicted R^2^ values (difference < 0.2) denotes the robustness and predictive capability of the developed response surface models (Equations (9) and (10)).

SF = +0.0928 − 0.0672 A − 0.0464 B − 0.0408 C − 0.0167 D + 0.0482 AB + 0.0119 AC+ 0.0647 AD  − 0.0047 BC − 0.0231 BD + 0.0243 CD − 0.0034 A^2^ + 0.0166 B^2^ + 0.0666 C^2^ (9)

Rn = +1.24 + 0.1489 A − 0.3394 B − 0.3417 C + 0.0850 D + 0.5212 AB + 0.0100 AC − 0.1200 AD  + 0.3850 BC − 0.0100 BD + 0.3888 CD + 0.2431 A^2^ + 0.1581 B^2^ + 0.0481 C^2^ + 0.1581 D^2^ (10)

**Figure 6:**
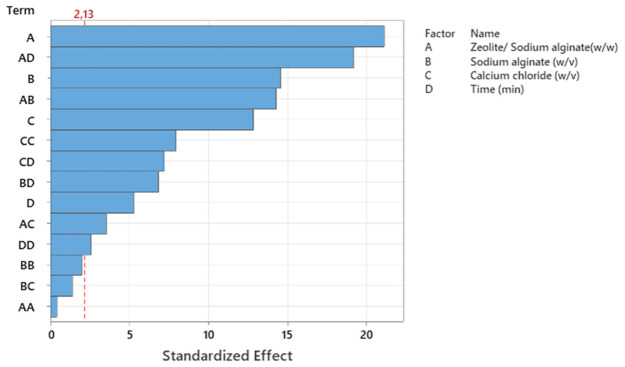
Pareto chart showing the standardized effects of process variables on sphericity factor (α = 0.05).

Although SF and Rn describe particle geometry, their statistical responses revealed distinct sensitivities to the processing parameters. For SF, all main effects (zeolite/sodium alginate ratio, sodium alginate concentration, calcium chloride concentration and processing time) were statistically significant (*p* < 0.0001), as seen in Table 2 and Figure 6. These findings indicate that sphericity is governed by both formulation composition and gelation dynamics [54]. Sphericity is largely established during droplet formation and early-stage ionotropic gelation, and it is therefore sensitive to formulation-driven viscosity changes and crosslinking kinetics [55]. The important contribution of interaction terms (AB and AD) is also reflected in Equation (9). Overall, these results show that sphericity is highly sensitive to coupled effects between material composition and processing conditions during droplet formation and early-stage ionic gelation.

**Figure 7:**
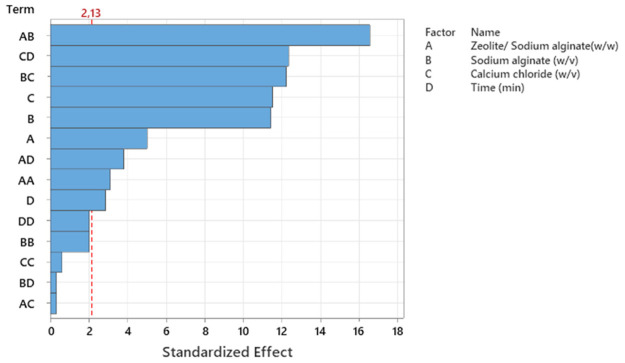
Pareto chart showing the standardized effects of process variables on roundness (α = 0.05).

In contrast, Rn was influenced by formulation-related parameters (sodium alginate concentration and calcium chloride concentration), while curing time exhibited a relatively weaker effect (Figure 7). This tendency is consistent with the regression coefficients reported in Equation (10). Roundness is linked to internal gel network uniformity, which is primarily governed by alginate–calcium interactions rather than curing time once gelation is established [55].

**Figure 8:**
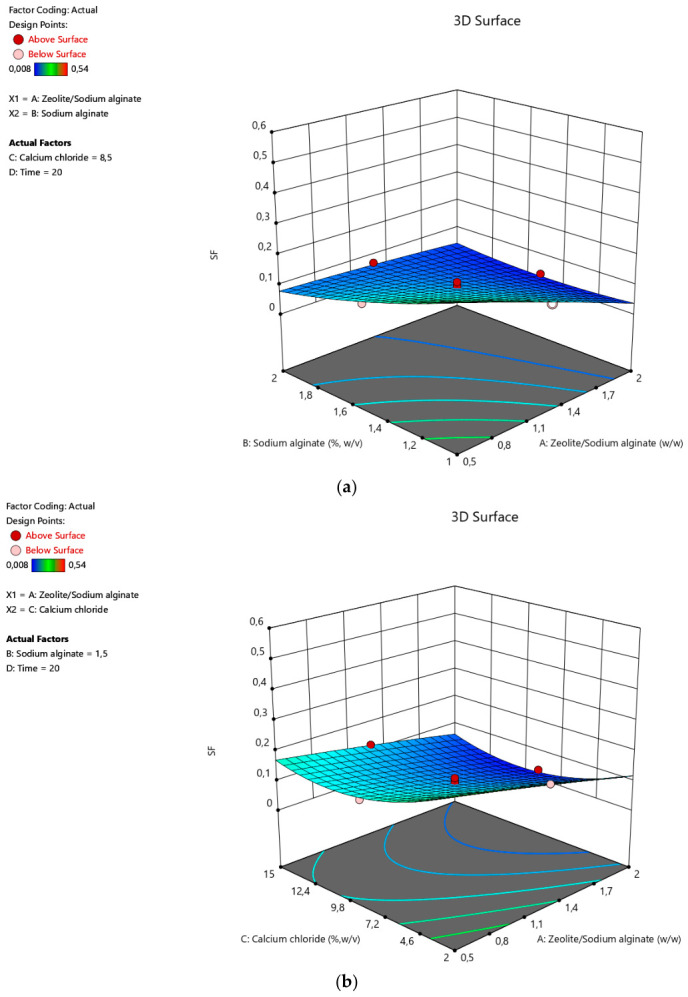

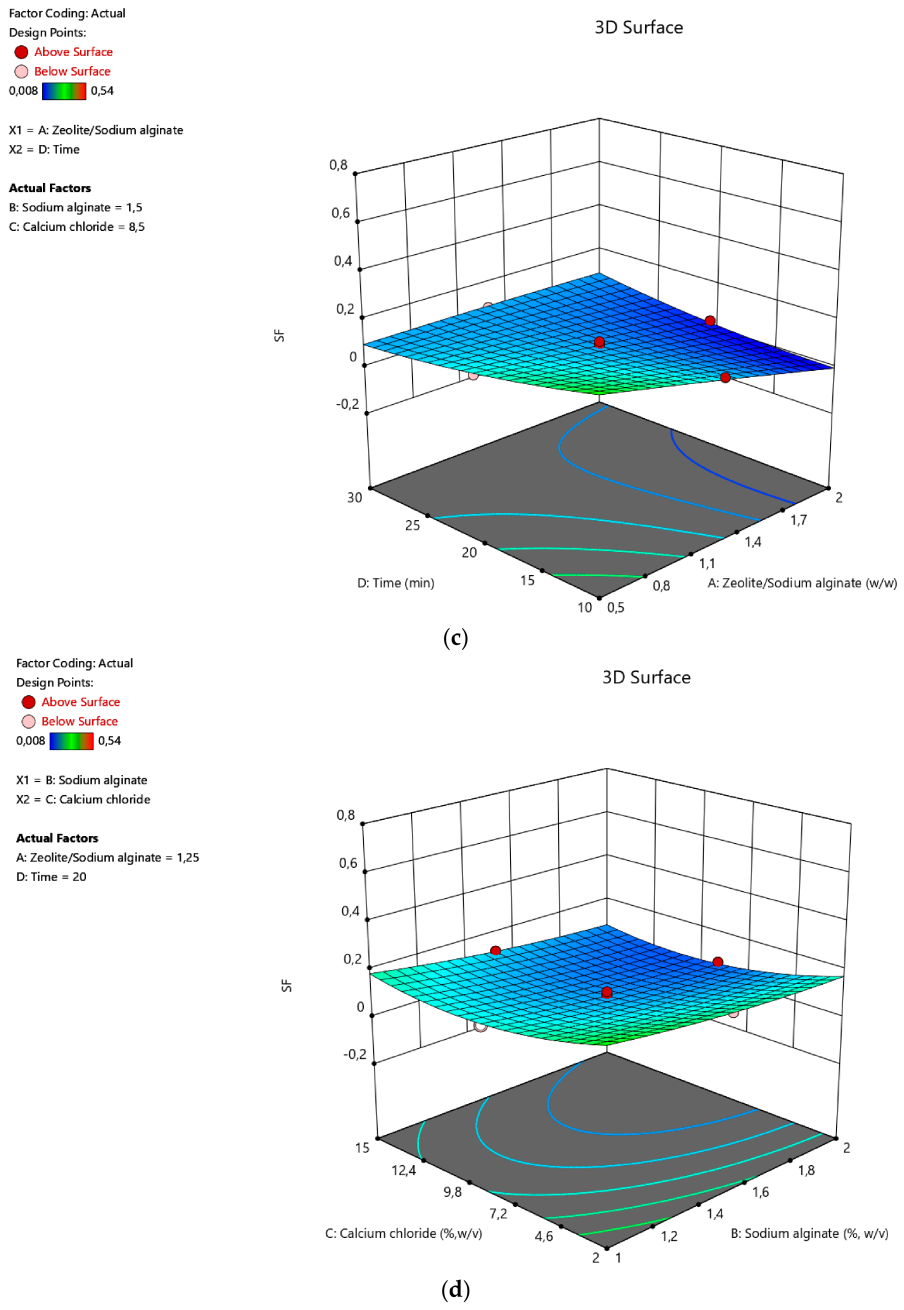
Three-dimensional response surface plots showing the interactive effects of zeolite/sodium alginate and sodium alginate (**a**), zeolite/sodium alginate and calcium chloride (**b**), zeolite/sodium alginate and time (**c**), and sodium alginate and calcium chloride (**d**) on sphericity factor of CNZ@AMOX–alginate microcapsules.

**Figure 9:**
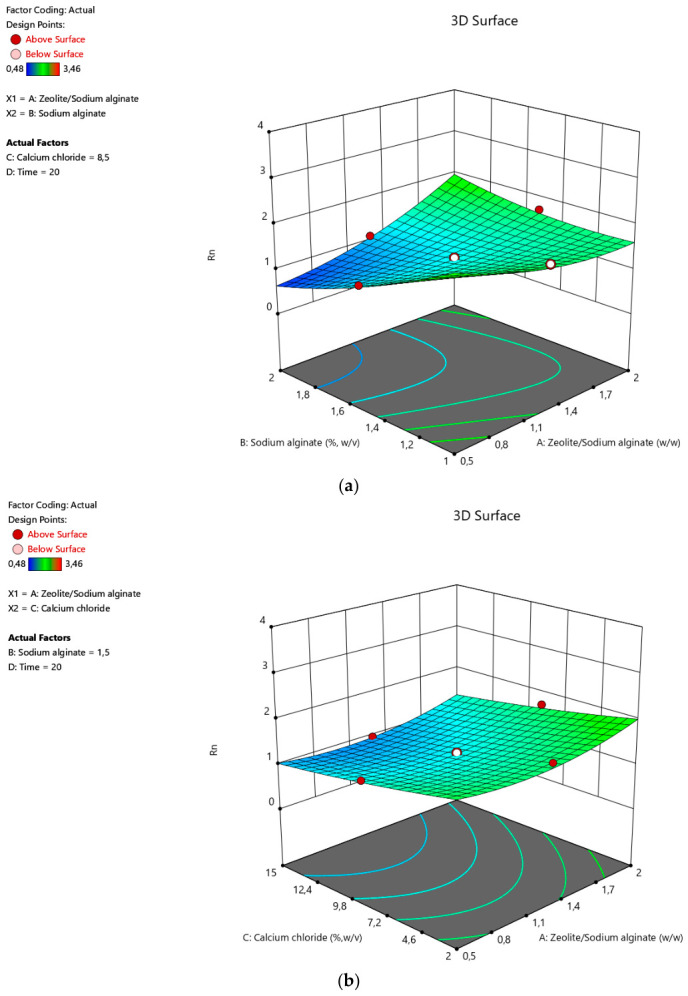

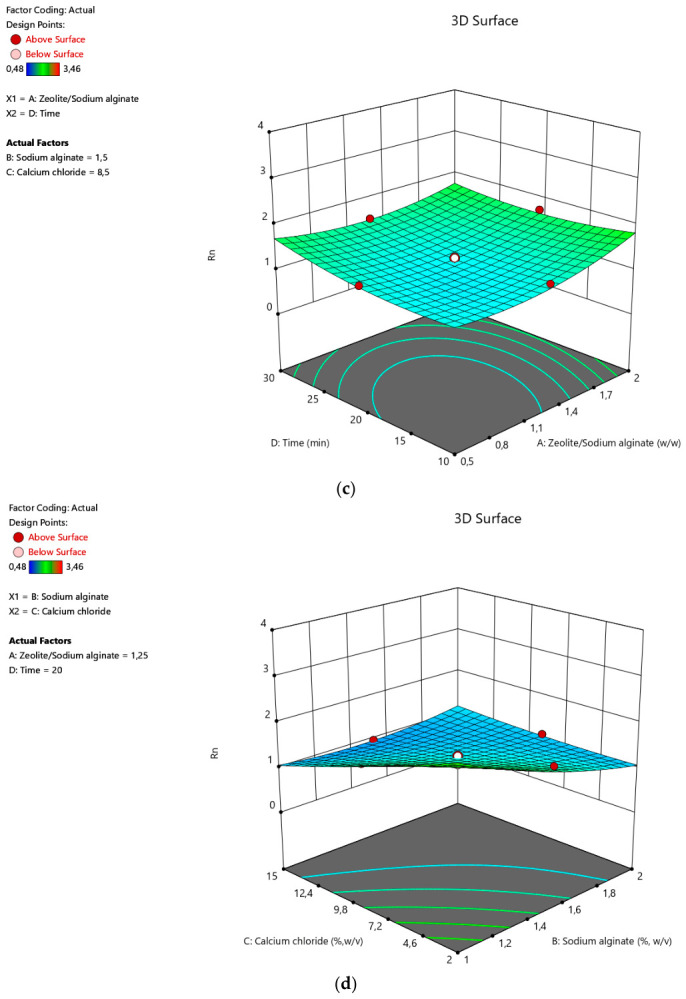
Three-dimensional response surface plots showing the interactive effects of zeolite/sodium alginate and sodium alginate (**a**), zeolite/sodium alginate and calcium chloride (**b**), zeolite/sodium alginate and time (**c**), and sodium alginate and calcium chloride (**d**) on roundness of CNZ@AMOX–alginate microcapsules.

Although both SF and Rn describe particle geometry, they reflect different structural levels of alginate microcapsules. Sphericity factor is mainly determined during droplet formation and the early stages of ionic gelation, making it sensitive to both formulation composition and processing conditions such as curing time. Conversely, roundness depends on the internal gel network uniformity and polymer–calcium interactions, which are formed after gelation is completed. Therefore, Rn exhibits a stronger dependence on formulation-related parameters and a weaker sensitivity to processing time, explaining the distinct statistical behavior observed for these two geometric descriptors. To illustrate the interaction effects of formulation and processing variables on particle geometry, the three-dimensional response surface plots (Figure 8 and Figure 9) were generated using Design-Expert software (Version 12.0.1.0, Stat-Ease Inc., Minneapolis, MN, USA).

The general trend of Figure 8 is consistent with Equation (9), where SF tends to decrease as A and B increase (negative linear terms). However, the interactions of AB (+) and AD (+) can partially balance the effect of B or time at certain levels of A. This indicates that the slope on the surfaces is not perfectly parallel but has an interactive structure [56].

As seen in Figure 8a, SF decreases as zeolite/alginate ratio and alginate concentration increase. This is consistent with the negative A and B main effects in Equation (9). The positive AB term suggests that the negative effect of B varies depending on the level of A. Compositional changes determine sphericity by affecting the viscosity, flow behavior, and early gelation during droplet formation [54,55]. Figure 8b shows that SF tends to decrease (−0.0408 C in Equation (9)) as CaCl_2_ increases. Since the interaction between these two variables is small and positive, the effect of CaCl_2_ may vary slightly with the level of A. However, the whole direction remains negative. This can be explained by mechanisms such as excessive/sudden formation of the outer gel layer and local shrinkage/deformation on the surface [54].

On the other hand, the tendency of SF to decrease as D (duration) increases is consistent with −0.0167 D in Equation (9) (Figure 8c). However, the positive AD explains that the negative effect of duration is partially compensated for at high A levels (the surface becoming flatter in some regions). This can be related to the early locking of the shape and the subsequent maturation of the network’s internal structure [55]. The interaction between alginate and calcium solution can be seen in Figure 8d. Since both B and C have negative linear effects, SF decreases slightly when they increase together. However, since Equation (9) contains B^2^ (+) and C^2^ (+) terms, a curvature-dependent minimum/plateau behavior can be observed in some ranges. That is why the surface appears curved instead of a flat plane [56].

For roundness, the general trend of the parameters is consistent with Equation (10) as seen in Figure 9. Rn tends to increase as A (zeolite level) and D (time) increase (positive A and D), while it decreases as B (alginate) and C (calcium chloride) increase (negative B and C). Furthermore, strong interactions such as AB (+) and BC (+) reveal that Rn exhibits multivariable interaction effects rather than a univariate response [56].

Figure 9a shows the interaction between zeolite and alginate. Due to the positive coefficient of A and the negative coefficient of B, Rn generally increases as A increases and decreases as B increases. The strong positive AB term in Equation (10) explains why the negative effect of B might decrease in certain regions when A is high (or conversely, the effect of B might be sharper when A is low). On the other hand, Figure 9b shows the interaction between zeolite and calcium levels. Due to the main negative effect of C, Rn tends to decrease as CaCl_2_ increases. Rn increases as A increases. The positive CD (and the very small AC) might lead to the effect of A appearing more dominant in some regions.

As seen in Figure 9c, Rn tends to increase as A and time increase since A and D are positive. However, the negative AD indicates that “increased time” does not provide the same benefit in every region depending on A (the benefit of increased time may decrease at high A). For the interaction between alginate and calcium chloride (Figure 9d), the positive BC causes the surface to be less steep than expected when the two variables change together (smoother transitions in some regions), while the negative main effects of B and C decrease Rn. This supports the idea that internal network homogeneity and crosslinking distribution are controlled together in a way that cannot be explained by a single variable [55]. These findings are consistent with very recent studies demonstrating that alginate and calcium chloride concentrations significantly influence the sphericity factor, roundness, and the whole morphology of alginate-based microcapsules due to their direct effect on ionic crosslinking and gel network formation [57,58,59,60].

### 3.5. Multi-Response Optimization and Validation

**Figure 10:**
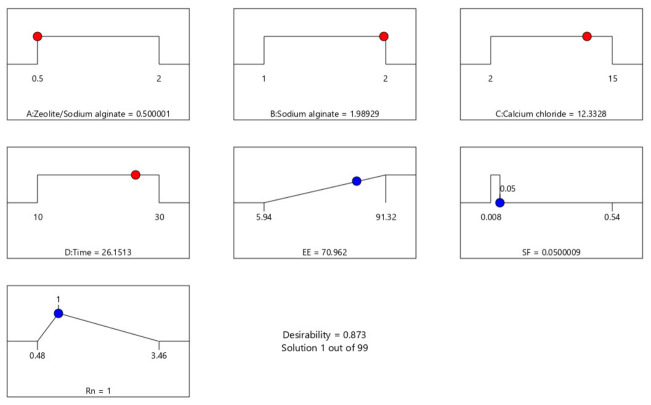
Desirability ramps plot showing the optimization of encapsulation efficiency, sphericity factor and roundness of CNZ@AMOX–alginate microcapsules. Red dots indicate the optimized levels of the independent variables (A–D), while blue dots indicate the predicted optimal response values (EE, SF and Rn).

Multi-response optimization was performed using the desirability function in Design-Expert software to determine the optimal processing conditions for CNZ@AMOX–alginate microcapsules. The optimization aimed to maximize EE and Rn while minimizing SF, thereby producing microcapsules with high encapsulation performance and desirable geometric properties. The ramp plots displaying the individual desirability functions and the optimal levels of the independent variables are presented in Figure 10.

As shown in Figure 10, the optimal formulation conditions were determined as zeolite/sodium alginate ratio of 0.500, sodium alginate concentration of 1.989% (*w*/*v*), CaCl_2_ concentration of 12.333% (*w*/*v*), and curing time of 26.151 min, with an overall desirability value of 0.873. Under these optimized conditions, the predicted responses were 70.962% for EE, 0.05 for SF and 1 for Rn. Experiments performed under the optimized conditions yielded values of 70.312% for EE, 0.0489 for SF, and 0.989 for Rn, which were in close agreement with the predicted values. The differences between the experimental and predicted responses were ≤2%, confirming satisfactory model performance. Overall, optimization of alginate concentration, calcium chloride concentration, zeolite ratio, and curing time was essential for achieving high encapsulation efficiency and desirable microcapsule morphology. Celli et al. similarly evaluated calcium chloride concentration, sodium alginate concentration, and bead-hardening time during optimization of anthocyanin encapsulation in alginate microparticles using a Box–Behnken design [61]. Alkhatib et al. evaluated calcium chloride concentration and curing time using a 3^2^ full factorial design for encapsulation of black seed oil in alginate beads [62]. Najafi-Soulari et al. optimized the lemon balm antioxidant encapsulation in alginate beads with three factors (CaCl_2_, alginate and active substance concentrations) through response surface methodology [63].

To conclude, the optimized CNZ@AMOX–alginate microcapsules exhibited an average particle diameter of approximately 101 µm (calculated from d_min_ and d_max_ measurements), together with a sphericity factor of 0.0489 and a roundness value of 0.989, indicating the successful formation of nearly spherical microcapsules.

### 3.6. In Vitro Release Kinetics and Mechanistic Interpretation

**Figure 11:**
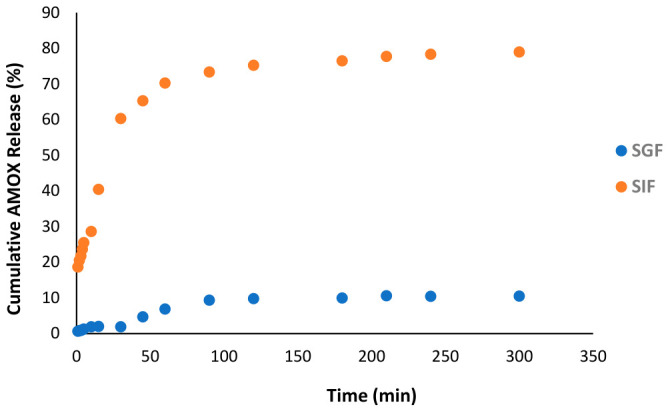
Cumulative AMOX release profiles from CNZ@AMOX–alginate microcapsules in simulated gastric fluid (SGF, pH 1.2) and simulated intestinal fluid (SIF, pH 6.8).

Figure 11 illustrates the cumulative release profiles of AMOX from the optimized CNZ@AMOX–alginate microcapsules under SGF and SIF conditions. A pronounced pH-dependent release behavior was observed. During the gastric stage (SGF), AMOX release remained limited, reaching only 10.47% after 300 min, indicating that the calcium–alginate network effectively restricted drug diffusion under acidic conditions. In contrast, substantially higher release was observed in SIF, where the cumulative release reached 78.93% after 300 min. The accelerated release under intestinal conditions can be attributed to the partial relaxation of the alginate network and the enhanced diffusion of AMOX at near-neutral pH. These findings demonstrate that the developed hybrid microcapsules effectively protect the drug in the gastric environment while promoting its release in the intestinal medium, supporting their potential as pH-responsive oral delivery systems.

The release data were fitted to zero-order, first-order, Higuchi and Korsmeyer–Peppas kinetic models. The corresponding linear regression plots are provided in Figures S2–S5 (Supplementary Materials), while the calculated kinetic parameters are summarized in Table 3.

As shown in Table 3, the Korsmeyer–Peppas model provides the best fit for release in SGF (R^2^ = 0.9556), followed by the Higuchi model (R^2^ = 0.9210). The zero-order and first-order models show poorer fits (R^2^ ≈ 0.80). These results indicate that drug release in the acidic medium is primarily governed by diffusion-controlled transport through the alginate network. The release exponent (n = 0.5686) suggests a non-Fickian (anomalous) transport mechanism, indicating that both diffusion and polymer relaxation/swelling phenomena contribute to drug release [41,64]. The relatively low kinetic constants (k_0_ = 0.0004 mg min^−1^; k_1_ = 0.0004 min^−1^; k_H_ = 0.0073 mg min^−1/2^) confirm the limited drug release in SGF and the stability of the calcium–alginate gel network under acidic conditions [13].

In the case of SIF, the Korsmeyer–Peppas model gives the best fit (R^2^ = 0.8985), while the Higuchi, first-order, and zero-order models produce lower fits (R^2^ = 0.8369, R^2^ = 0.7410, R^2^ = 0.6445, respectively). Additionally, the release exponent of the Korsmeyer–Peppas model in SIF (n = 0.3364) corresponds to a Fickian diffusion mechanism [64,65]. In the intestinal environment, release is almost entirely diffusion controlled. Although the alginate matrix was expected to swell more at pH 6.8 due to the partial dissolution of the Ca^2+^ cross-links, the presence of the zeolite–AMOX hybrid and the high crosslinking density (12.3% CaCl_2_ used in optimization) suppressed matrix relaxation. Consequently, the contribution of swelling decreased, while diffusion became dominant. The higher Higuchi constant (k_H_ = 0.0412 mg min^−1/2^) and increased first-order constant (k_1_ = 0.0048 min^−1^) also indicate accelerated release under intestinal conditions.

In summary, the suppression of the relaxation contribution in SIF following zeolite incorporation (n = 0.3364) resulted in a more predictable and diffusion-driven intestinal release. This behavior is consistent with previous studies of alginate matrix systems, in which the Korsmeyer–Peppas model frequently provided the best fit for release kinetics [64,66,67].

## 4. Conclusions

Calcium–alginate microcapsules containing natural clinoptilolite–amoxicillin hybrids were successfully developed using response surface methodology. Encapsulation efficiency and particle geometry were significantly influenced by the calcium chloride concentration and the zeolite/alginate ratio, highlighting the importance of crosslinking density and compositional balance in controlling microcapsule formation. The statistical models showed high predictive capability (R^2^ > 0.98), and multi-response optimization identified conditions that provided high encapsulation efficiency and desirable microcapsule morphology. Drug release was limited under simulated gastric (SGF) conditions but increased substantially in simulated intestinal fluid (SIF), demonstrated the pH-dependent behavior of the system and its potential to protect AMOX in the gastric environment, while promoting its release in the intestine. Moreover, incorporation of the clinoptilolite-AMOX hybrid reduced the burst release and supported a more controlled release profile Collectively, these findings demonstrate the potential of clinoptilolite-containing calcium/alginate microcapsules as an effective oral delivery platform for the site-selective and controlled release of AMOX.

## Figures and Tables

**Table 1:** Experimental design matrix and corresponding experimental responses (encapsulation efficiency, sphericity factor, and roundness) for the microcapsules prepared by ionic gelation *.

Run	A:Zeolite/Sodium Alginate (*w*/*w*)	B:Sodium Alginate (%, *w*/*v*)	C:Calcium Chloride (%, *w*/*v*)	D:Time (min)	EE (%)	SF	Rn
1	2/10	1	2	10	9.14	0.16 ± 0.02	3.02 ± 0.01
2	1/20	1	2	30	32.6	0.27 ± 0.04	3.19 ± 0.05
3	1/10	1.5	8.5	20	35.8	0.10 ± 0.02	1.22 ± 0.02
4	1/10	1.5	2	20	13.6	0.20 ± 0.04	1.75 ± 0.04
5	1/20	2	2	30	17.8	0.15 ± 0.03	0.59 ± 0.04
6	2/10	1	15	30	34.1	0.16 ± 0.03	1.51 ± 0.03
7	1/20	1	15	10	76.5	0.38 ± 0.01	1.08 ± 0.02
8	1/20	2	15	30	71.7	0.07 ± 0.05	1.36 ± 0.01
9	1/10	1.5	8.5	20	29.6	0.11 ± 0.02	1.22 ± 0.02
10	2/10	2	15	30	24.1	0.18 ± 0.04	2.59 ± 0.02
11	2/10	2	15	10	29.0	0.01 ± 0.04	1.80± 0.03
12	1/10	1.5	8.5	20	28.8	0.09 ± 0.02	1.15 ± 0.03
13	1/10	1.5	8.5	30	27.9	0.09 ± 0.02	1.51 ± 0.04
14	2/10	1	15	10	10.4	0.04 ± 0.03	0.76 ± 0.02
15	2/10	2	2	10	17.4	0.11 ± 0.02	2.50 ± 0.02
16	1/20	1.5	8.5	20	76.0	0.15 ± 0.02	1.38 ± 0.01
17	2/10	1	2	30	12.8	0.14 ± 0.01	1.93 ± 0.01
18	1/20	1	15	30	91.3	0.24 ± 0.01	2.49 ± 0.04
19	1/10	1	8.5	20	20.0	0.15 ± 0.05	1.83 ± 0.05
20	1/10	1.5	8.5	20	29.5	0.09 ± 0.02	1.20± 0.02
21	1/20	2	15	10	91.1	0.15 ± 0.03	0.48 ± 0.02
22	1/20	1	2	10	38.3	0.54 ± 0.03	3.46 ± 0.01
23	1/10	1.5	8.5	10	26.1	0.14 ± 0.02	1.42 ± 0.02
24	2/10	1.5	8.5	20	34.9	0.03 ± 0.04	1.72 ± 0.02
25	1/10	1.5	8.5	20	28.0	0.09 ± 0.04	1.27 ± 0.04
26	1/10	1.5	15	20	38.2	0.12 ± 0.01	0.96 ± 0.02
27	1/10	2	8.5	20	19.6	0.07 ± 0.01	1.10 ± 0.02
28	1/20	2	2	10	45.6	0.30 ± 0.04	0.93 ± 0.03
29	2/10	2	2	30	5.9	0.22 ± 0.06	1.81 ± 0.02
30	1/10	1.5	8.5	20	27.4	0.07 ± 0.04	1.18 ± 0.02

* Data are given as the mean (n = 3) ± standard deviation.

**Table 2:** Analysis of variance findings on encapsulation efficiency, sphericity factor and roundness, respectively.

	Source	Sum of Squares	df	Mean Square	F-Value	*p*-Value	
**EE**	**Model**	15,702.19	14	1121.58	128.48	<0.0001	significant
	A-Zeolite/Sodium alginate	7324.94	1	7324.94	839.07	<0.0001	
	B-Sodium alginate	0.4640	1	0.4640	0.0532	0.8208	
	C-Calcium chloride	4146.87	1	4146.87	475.02	<0.0001	
	D-Time	35.98	1	35.98	4.12	0.0605	
	AB	32.01	1	32.01	3.67	0.0748	
	AC	1295.46	1	1295.46	148.39	<0.0001	
	AD	150.49	1	150.49	17.24	0.0009	
	BC	5.75	1	5.75	0.6584	0.4298	
	BD	625.63	1	625.63	71.67	<0.0001	
	A^2^	192.31	1	192.31	22.03	0.0003	
	B^2^	1675.09	1	1675.09	191.88	<0.0001	
	C^2^	270.25	1	270.25	30.96	<0.0001	
	D^2^	43.62	1	43.62	5.00	0.0410	
	**Residual**	23.99	1	23.99	2.75	0.1181	
	Lack of Fit	130.95	15	8.73			
	Pure Error	84.19	10	8.42	0.9003	0.5867	not significant
	**Cor Total**	46.76	5	9.35			
	C.V.: 8.50%	R^2^ = 0.9917		Adjusted R^2^ = 0.9840		Predicted R^2^ = 0.9593	
**SF**	**Model**	0.3435	14	0.0245	134.47	<0.0001	significant
	A-Zeolite/Sodium alginate	0.0812	1	0.0812	445.07	<0.0001	
	B-Sodium alginate	0.0387	1	0.0387	212.30	<0.0001	
	C-Calcium chloride	0.0300	1	0.0300	164.49	<0.0001	
	D-Time	0.0050	1	0.0050	27.59	<0.0001	
	AB	0.0372	1	0.0372	203.63	<0.0001	
	AC	0.0023	1	0.0023	12.50	0.0030	
	AD	0.0670	1	0.0670	366.95	<0.0001	
	BC	0.0004	1	0.0004	1.93	0.1854	
	BD	0.0085	1	0.0085	46.64	<0.0001	
	A^2^	0.0095	1	0.0095	51.84	<0.0001	
	B^2^	0.0000	1	0.0000	0.1653	0.6900	
	C^2^	0.0007	1	0.0007	3.91	0.0668	
	D^2^	0.0115	1	0.0115	62.96	<0.0001	
	**Residual**	0.0012	1	0.0012	6.62	0.0212	
	Lack of Fit	0.0027	15	0.0002			
	Pure Error	0.0019	10	0.0002	1.05	0.5091	not significant
	**Cor Total**	0.0009	5	0.0002			
	C.V.: 8.79%	R^2^ = 0.9921		Adjusted R^2^ = 0.9847		Predicted R^2^ = 0.9555	
**Rn**	**Model**	16.22	14	1.16	72.90	<0.0001	significant
	A-Zeolite/Sodium alginate	0.3990	1	0.3990	25.11	0.0002	
	B-Sodium alginate	2.07	1	2.07	130.53	<0.0001	
	C-Calcium chloride	2.10	1	2.10	132.25	<0.0001	
	D-Time	0.1301	1	0.1301	8.19	0.0119	
	AB	4.35	1	4.35	273.61	<0.0001	
	AC	0.0016	1	0.0016	0.1007	0.7554	
	AD	0.2304	1	0.2304	14.50	0.0017	
	BC	2.37	1	2.37	149.26	<0.0001	
	BD	0.0016	1	0.0016	0.1007	0.7554	
	A^2^	2.42	1	2.42	152.19	<0.0001	
	B^2^	0.1531	1	0.1531	9.63	0.0073	
	C^2^	0.0647	1	0.0647	4.07	0.0618	
	D^2^	0.0060	1	0.0060	0.3768	0.5485	
	**Residual**	0.0647	1	0.0647	4.07	0.0618	
	Lack of Fit	0.2383	15	0.0159			
	Pure Error	0.2152	10	0.0215	4.66	0.0516	not significant
	**Cor Total**	0.0231	5	0.0046			
	C.V.: 7.84%	R^2^ = 0.9855		Adjusted R^2^ = 0.9720		Predicted R^2^ = 0.9089	

**Table 3:** Kinetic model parameters for the release of AMOX from CNZ@AMOX–alginate microcapsules.

GI System	Zero-Order Kinetic Model		First-Order Kinetic Model		Higuchi Model		Korsmeyer–Peppas Model		
	k_0_ (mg dk^−1^)	R^2^	k_1_ (dk^−1^)	R^2^	k_H_ (mg dk^−1/2^)	R^2^	n	K (dk^−n^)	R^2^
SGF	0.0004	0.8042	0.0004	0.8086	0.0073	0.9210	0.5686	0.0050	0.9556
SIF	0.002	0.6445	0.0048	0.7410	0.0412	0.8369	0.3364	0.159	0.8985

## Data Availability

The original contributions presented in this study are included in the article/Supplementary Materials. Further inquiries can be directed to the corresponding authors.

## Supplementary Materials

The following supporting information can be downloaded at https://www.mdpi.com/article/10.3390/pharmaceutics18070878/s1, Figure S1: Preparation of CNZ@AMOX–alginate microcapsules; Figure S2: Zero-order kinetic model fitting for AMOX release from CNZ@AMOX–alginate microcapsules in simulated gastric fluid (SGF) and simulated intestinal fluid (SIF); Figure S3: First-order kinetic model fitting for AMOX release from CNZ@AMOX–alginate microcapsules in SGF and SIF; Figure S4: Higuchi model fitting for AMOX release from CNZ@AMOX–alginate microcapsules in SGF and SIF; Figure S5: Korsmeyer–Peppas model fitting for AMOX release from CNZ@AMOX–alginate microcapsules in SGF and SIF; Table S1. Independent variables, their coded levels and response constraints used in the face-centered central composite design for optimization of CNZ@AMOX–alginate microcapsules prepared by ionic gelation; Table S2. HPLC operating conditions and gradient elution program for determination of amoxicillin concentration.
