# Supplementary material for: Natural Clinoptilolite as a Functional Mineral Component in Alginate Hybrid Microcapsules for Controlled Amoxicillin Release
Source: Pharmaceutics. 2026 Jul 17;18(7):878. doi: 10.3390/pharmaceutics18070878 (PMC13415116; doi:10.3390/pharmaceutics18070878)
Supplement: Supplementary file 1 [file pharmaceutics-18-00878-s001.zip › pharmaceutics-4416039-supplementary.pdf]

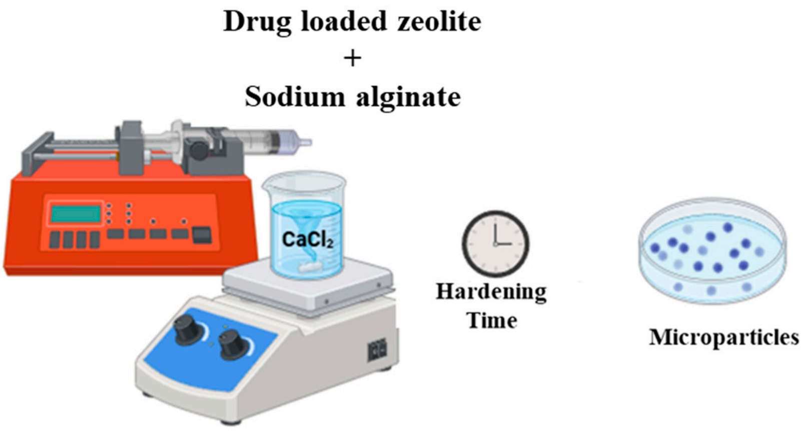

Figure S1. Preparation of CNZ@AMOX-alginate microcapsules.

Table S1. Independent variables, their coded levels and response constraints used in the face centered central composite design for optimization of CNZ@AMOX-alginate microcapsules prepared by ionic gelation.

**Independent variable**	**Coded levels**		
	**−1**	**0**	**1**
**A:** Zeolite/Sodium alginate(w/w)	1/20	1/10	2/10
**B:** Sodium alginate (%, w/v)	1	1.5	2
**C:** Calcium chloride (%, w/v)	2	8.5	15
**D:** Time (min)	10	20	30
**Dependent variable**	Constraints		
Y_1: EE (%)	Maximize		
Y_2: SF	Minimize		
Y_3: Rn	Maximize		

Table S2. HPLC operating conditions and gradient elution program for determination of amoxicillin concentration.

**HPLC Conditions**	**Program**		
**Model:** UV-Tech HPLC 1511 (UVTech Inc, Beijing, China)	**Time (min)**	**A (%)**	**B (%)**
**Colon:** Luster C18 (250.0 × 4.6 mm; 5 µm)	0.0	90	10
**Mobile phase:** A = Water + % 0.1 formic acid (v/v)	3.0	75	25
B = Acetonitrile + % 0.1 formic acid (v/v)	4.5	50	50
**Detection wavelength:** 225 nm	6.0	25	75
**Flow rate:** 1.8 mL/min	7.5	0	100
**Column temperature:** 40 °C	8.5	90	10
**Injection volume:** 5 µL	10	90	10

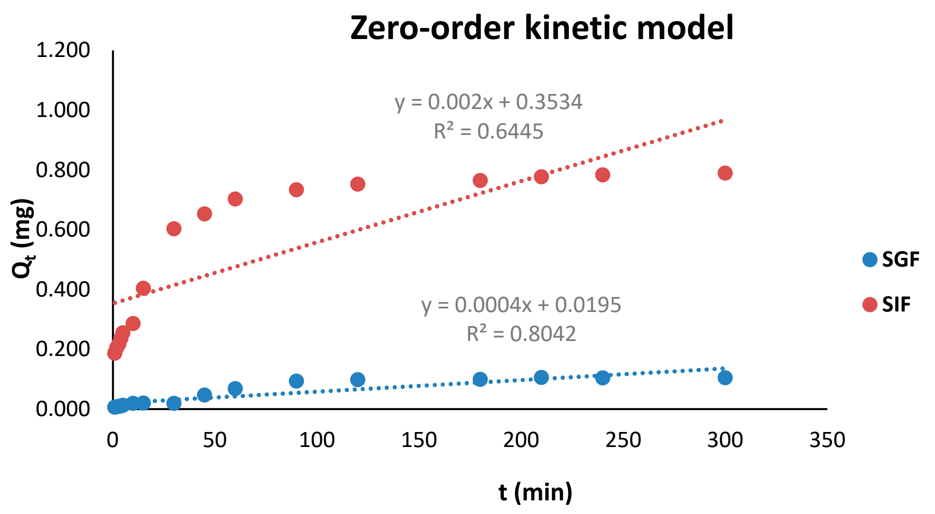

Figure S2. Zero-order kinetic model fitting for AMOX release from CNZ@AMOX-alginate microcapsules in simulated gastric fluid (SGF) and simulated intestinal fluid (SIF).

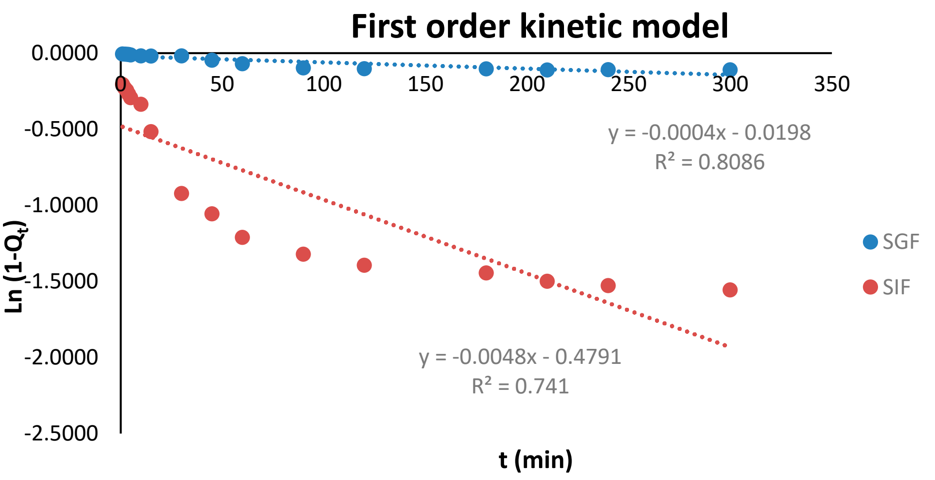

Figure S3. First-order kinetic model fitting for AMOX release from CNZ@AMOX-alginate microcapsules in SGF and SIF.

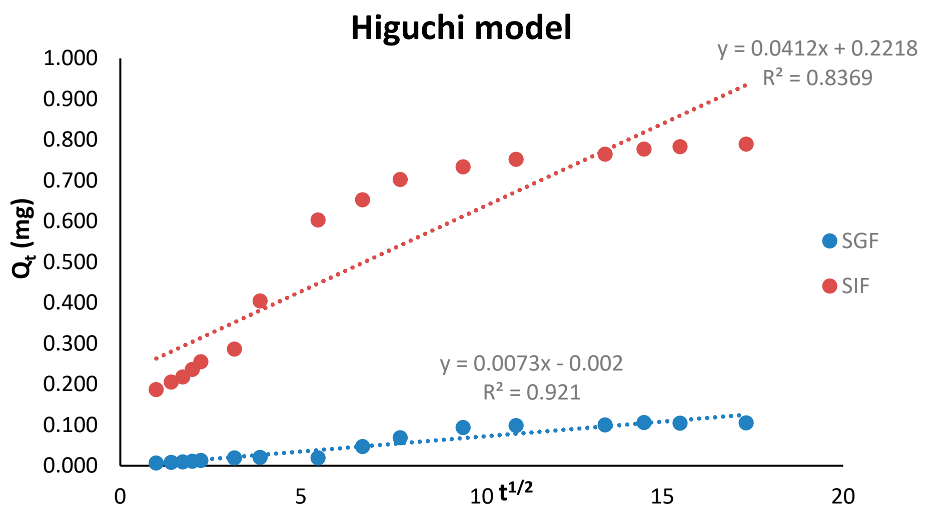

Figure S4. Higuchi model fitting for AMOX release from CNZ@AMOX-alginate microcapsules in SGF and SIF.

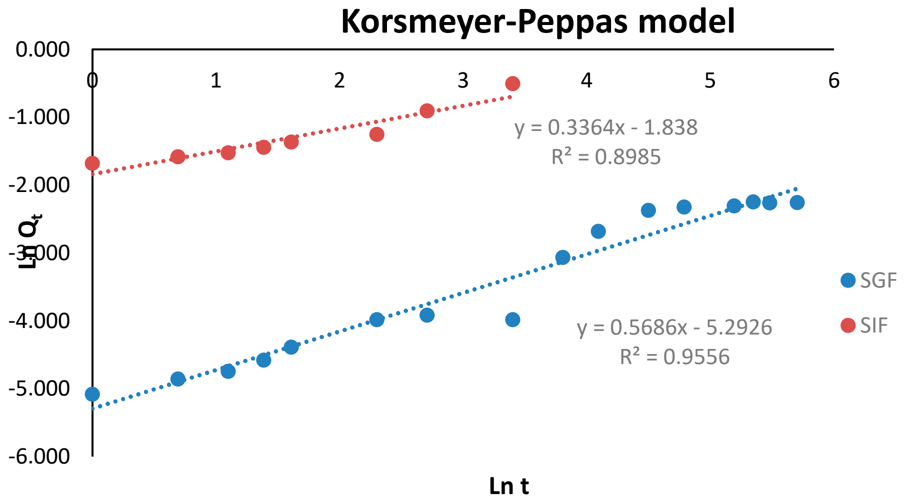

Figure S5. Korsmeyer-Peppas model fitting for AMOX release from CNZ@AMOX-alginate microcapsules in SGF and SIF.
